# Patterns of international collaboration in cardiovascular research in sub-Saharan Africa

**DOI:** 10.5830/CVJA-2015-082

**Published:** 2016

**Authors:** Remare Ettarh

**Affiliations:** Alberta Innovates – Health Solutions, Edmonton, Canada

**Keywords:** cardiovascular research, Africa, collaboration, co-authorship analysis, academic impact

## Abstract

The rising prevalence of cardiovascular disease in sub-Saharan Africa (SSA) constitutes a significant health and socio-economic challenge for the countries in the region. This study examines the patterns and scientific impact of international collaboration in cardiovascular research (CVR) in SSA. Bibliographic data from 2005 to 2014 were obtained from the Web of Science for cardiovascular-related publications with at least one author affiliated to an SSA country. The number of publications involving multiple SSA countries over this period accounted for less than 10% of the total number of multicountry publications that included at least one SSA country. Collaboration patterns reflected dominance by countries in Europe and North America, with South Africa accounting for the bulk of scientific collaboration in CVR within SSA. The findings indicate that pro-active strategies are needed to strengthen collaboration in CVR across SSA for the region to derive health and socio-economic benefits from locally conducted research.

## Abstract

The increasing burden of cardiovascular diseases (CVD) in sub-Saharan Africa has been reported in recent studies on the global burden of disease.^1^ The epidemiological transition in the region from a predominantly infectious disease burden to one that includes a high prevalence of non-communicable conditions, such as cardiovascular diseases, poses significant challenges, not only for the economies and health systems of the countries, but also for the local health research sector, which often lacks the capacity to drive knowledge advancement and innovation leading to positive health impacts.^2^

Over the last decade, there have been efforts to develop capacity in developing countries. These strategies have included promoting international collaboration through the joint development of projects by research groups in developed and developing countries.^3^,^4^ The value of this collaborative approach for the developing country partner lies in the availability of financial and technical resources for research, opportunities to develop and nurture institutional research capacity, and the potential impact of locally focused research on the health system.

The negligible contribution of Africa to global scientific knowledge and impact is well documented.5 However, there has been a gradual increase in the volume of scientific publications that include authorship from Africa in recent years, albeit with a substantial dominance by non-African co-authors.^5^,^6^ Some of the reasons for this pattern of scientific output include: (1) low budgetary funding for research by African countries; (2) lack of investment in scientific infrastructure and research equipment; (3) emphasis on personnel development within the higher education system rather than on support for research; and (4) the dearth of skilled research scientists in the region, partly associated with the brain drain phenomenon. There are suggestions that these challenges can be partly addressed by increased international collaboration, which reduces research cost and time, enhances knowledge transfer across borders, and improves scientific impact.^7^

The extent of research collaboration across SSA has been the subject of a few recent publications,^5^,^8^,^9^ although little is known about the patterns of collaboration within the area of CVR in this region. Additional studies are required to understand the extent of scientific output as well as the patterns and impact of collaboration in CVR among countries in SSA. The evidence from such studies would be valuable in developing strategies to advance knowledge in cardiovascular disease prevention and care in the region.

The use of co-authorship networks for analysing research collaboration at the individual or micro-level is well established,^10^,^11^ although there is an upward trend in its application for studying macro-level or international collaboration.^12^ Network analysis offers possibilities for visualising and understanding the relationships between the collaborating research entities. The methodologies allow for the identification of hubs, boundary spanners, clusters and the strength of relationships.^13^ Temporal analysis of the collaborative patterns is also possible using timesliced data.^14^ In a macro-level network, the vertices represent countries and the edges between them represent the presence of collaboration in the form co-authorship by one or more research groups. The weight of the edge reflects the number of publications by a pair of vertices or countries.

This study examines the indexed scientific output of cardiovascular research conducted solely or in part by researchers in sub-Saharan Africa over the last 10 years in order to analyse macro-level collaboration in the region. Specifically, the study uses citation and network analyses to: (1) examine the trends in scientific output and collaborative research patterns in CVR in SSA; (2) identify the top countries in SSA involved in collaborative CVR within the region and globally; and (3) assess the scientific impact of collaborative CVR in SSA.

## Methods

Bibliographic data were sourced from the Web of Science of Thompson Reuters. A search procedure was developed to retrieve the relevant publications for analysis. The databases included in the search included: Science Citation Index Expanded (SCI-EXPANDED), Conference Proceedings Citation Index – Science (CPCI-S), Conference Proceedings Citation Index – Social Science & Humanities (CPCI-SSH) and Arts & Humanities Citation Index (A&HCI). The publication period was restricted to 2005 to 2014. A set of key words for cardiovascular research was used for the search of the title, keyword and abstract fields.^15^ The list of the 47 countries that constitute sub-Saharan Africa was obtained from the World Bank^16^ and used as part of the advanced search protocol. Citation data were obtained for all the retrieved records by using the Create Citation Report function in Web of Science.

Pre-processing of the Web of Science data was done in Microsoft Excel. Country names were extracted from each author’s affiliation and saved with accompanying attribute data, such as year and type of publication. The resulting flat file was merged with citation data from Web of Science using unique identifiers for each publication. Duplicate country names, which occurred where co-authors were from the same country, were eliminated for each publication using macros within Microsoft Excel.

After cleaning, a total of 1 569 publications from 2005–2014 were available for analysis. The dataset was then split into two for subsequent citation and network analyses: one with single SSA country publications (n = 783) and another with multicountry publications (n = 786). The multi-country dataset was filtered to show only countries within SSA, to enable analysis of collaboration patterns within the region. For network analysis using this SSA-specific dataset, all publications with only one SSA country were excluded. A total of 75 publications were obtained, which included collaborations between multiple SSA countries.

## Data analysis

Research output was assessed using number of publications, disaggregated by region or country and by single or joint country authorship. The trend in research output was determined and illustrated over the 10-year period. The instances of collaboration were determined from adjacency matrices and used to determine the top 10 SSA countries involved in cardiovascular research collaboration and the top six countries outside the SSA with which these collaborations occurred. Citation analysis was done by examining the trend in citation rates in single-country- and multi-country-authored publications across the 10-year period.

Patterns of international collaboration in cardiovascular research involving SSA countries were analysed using traditional network analysis methodologies. The co-occurrence of countries in the affiliation field of publications was considered an instance of collaboration between the two countries. Co-authorship at the macro level is considered an effective approach to the analysis of research collaboration between countries.^10^

Preparation of datasets for network analysis involved creating adjacency matrices of countries reflecting instances of collaboration. Adjacency matrices were created using Visual Basic scripts run in Microsoft Excel, and used in the analysis of instances of collaboration within SSA. NodeXL, an open-source network analysis and visualisation application, was used for creating the network graph.^17^ Network data were uploaded into NodeXL as edge lists, i.e. a two-column list of country pairs that collaborated in publications. The network graph used to visualise international collaboration in CVR within SSA was created using the Harel-Koren fast multiscale algorithm.

## Results

## Cardiovascular research output

**Fig. 1.**
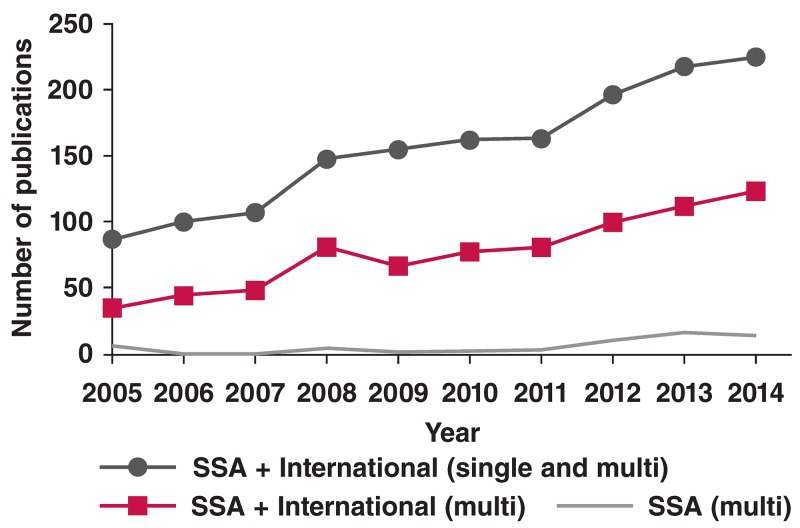
Trend in the number of publications in CVR with authorship from countries in SSA, 2005–2014. Single refers to publications with only one SSA country in its affiliations; multi refers to publications with more than one country in its affiliations but including at least one SSA country. International is used to refer to all countries outside SSA.

A total of 88 cardiovascular research publications with authorship in SSA were indexed in the Web of Science in 2005. There was a gradual increase to 225 publications in 2014. The trend for the fraction of CVR publications that involved multiple countries was from 37 in 2005 to 124 in 2014. There were eight CVR publications that involved multiple SSA countries in 2005, with a slight increase to 16 in 2014. Overall, the number of publications involving multiple SSA countries over the 10-year period accounted for less than 10% of the total number of multicountry publications that included at least one SSA country. The number of publications in the field with authorship from a single SSA country rose from 51 in 2005 to 101 in 2014 (not shown in Fig. 1). These data reflect minimal country-level collaboration in CVR in SSA and very limited growth in co-authorships across country borders over the last decade. The trend in cardiovascular research output in SSA is shown in Fig. 1.

Table 1 shows the cardiovascular research output by the top 10 countries in SSA and the percentage of publications that involved collaboration with other countries within or outside SSA. Eight of the 10 SSA countries had international collaborations on more than 50% of their publications. South Africa had the largest output over the decade with 1 016 publications, which was more than all the other SSA countries combined. Collaboration with other countries accounted for 50% of the output from South Africa, compared with 23% from Nigeria, which was the lowest in SSA. All of the publications from Zimbabwe involved collaboration with other countries.

**Table 1:** Demographic data of the study population (n = 118)

*Country*	*Total number of articles published*	*Number of articles that involved international collaboration*	*Percentage of articles that involved international collaboration*
South Africa	1016	507	50
Nigeria	228	52	23
Kenya	46	27	59
Cameroon	45	35	78
Uganda	37	30	81
Mozambique	35	31	89
Ghana	29	23	79
Senegal	29	13	45
Tanzania	17	15	88
Zimbabwe	12	12	100
Total	1494	745	50

## International collaboration in CVR in sub-Saharan Africa

Table 2 presents the extent of collaboration in CVR between the countries in SSA and the most frequent partner countries outside the region. The data reflect instances of collaboration between pairs of countries based on their co-occurrence in the author affiliations of a given publication. In network analysis terms, this is equivalent to the number of edges that any two countries share. The numbers in parentheses represent the percentage of the SSA country’s total instances of collaboration that involved the non-SSA country or all of SSA.

**Table 2:** Number of instances of cardiovascular research collaboration between the top 10 countries in sub-Saharan Africa and the most frequent non-SSA partner countries, 2005–2014

	*Total*	*USA (%)*	*England (%)*	*Italy (%)*	*France (%)*	*Germany (%)*	*Canada (%)*	*SSA (%)*
South Africa	1379	208 (15)	116 (8)	78 (6)	56 (4)	84 (6)	52 (4)	55 (4)
Nigeria	112	16 (14)	8 (7)	2 (2)	2 (2)	4 (4)	2 (2)	26 (23)
Cameroon	87	7 (8)	3 (3)	4 (5)	9 (10)	5 (6)	-	32 (37)
Uganda	82	17 (21)	10 (12)	7 (9)	4 (5)	3 (4)	3 (4)	12 (15)
Zimbabwe	76	1 (1)	5 (7)	2 (3)	1 (1)	–	4 (5)	16 (21)
Mozambique	61	5 (8)	6 (10)	-	14 (23)	1 (2)	2 (3)	9 (15)
Kenya	54	15 (28)	7 (13)	1 (2)	1 (2)	2 (4)	1 (2)	10 (19)
Ghana	51	8 (16)	4 (8)	-	2 (4)	5 (10)	-	16 (31)
Tanzania	36	4 (11)	6 (17)	1 (3)	1 (3)	-	-	8 (22)
Senegal	34	2 (6)	1 (3)	–	8 (24)	–	–	14 (41)

The numbers in parentheses represent the percentage of the SSA country’s total instances of collaboration that involved a non-SSA country or all of SSA.

South Africa recorded the highest instances of collaboration in SSA, with 15% of those occurring with the USA. Countries in SSA accounted for 4% of South Africa’s instances of collaboration in CVR. Nigeria had the second highest instances of collaboration in CVR, with the majority of these occurring with the USA. Cameroon and Senegal had most of their collaborations outside SSA with France, and the two countries also had the highest percentages of collaboration instances with SSA, at 37 and 41%, respectively.

Table 3 presents the instances of collaboration between the top 10 SSA countries in CVR. The numbers in parentheses represent the percentage of an SSA country’s collaborations within SSA that occurred with the paired SSA country. The highest number of instances of collaboration occurred between South Africa and Nigeria,^15^ and this accounted for 27% of South Africa’s SSA collaborations and 58% of Nigeria’s SSA collaborations. South Africa accounted for the majority of instances of collaboration for eight of the countries, with the exception of Senegal, which collaborated mostly with Cameroon.

**Table 3:** Number of instances of cardiovascular research collaboration between the top 10 countries in sub-Saharan Africa, 2005–2014

**	*South Africa(%)*	*Nigeria(%)*	*Cameroon(%)*	*Uganda(%)*	*Zimbabwe(%)*	*Mozambique(%)*	*Kenya(%)*	*Ghana(%)*	*Tanzania(%)*	*Senegal(%)*	*All SSA(%)*
South Africa		15 (27)	5 (9)	2 (4)	8 (15)	8 (15)	3 (5)	6 (11)	1 (2)	1 (2)	55
Nigeria	15 (58)		2 (8)	1 (4)	1 (4)	-	-	1 (4)	1 (4)	2 (8)	26
Cameroon	5 (16)	2 (6)		1 (3)	1 (3)	–	–	3 (9)	1 (3)	4 (13)	32
Uganda	2 (17)	1 (8)	1 (8)		1 (8)	–	1 (8)	–	1 (8)	-	12
Zimbabwe	8 (50)	1 (6)	1 (6)	1 (6)		-	-	-	1 (6)	-	16
Mozambique	8 (89)	-	-	-	-		-	-	-	-	9
Kenya	3 (30)	-	-	1 (10)	-	-		2 (20)	-	-	10
Ghana	6 (38)	1 (6)	3 (19)	-	-	-	2 (13)		-	-	16
Tanzania	1 (13)	1 (13)	1 (13)	1 (13)	1 (13)	-	-	-		-	8
Senegal	1 (7)	2 (14)	4 (29)	-	-	-	-	-	-		14

Numbers in parentheses are row percentages using the total number of instances of collaboration with all countries in SSA as denominator.

**Fig. 2.**
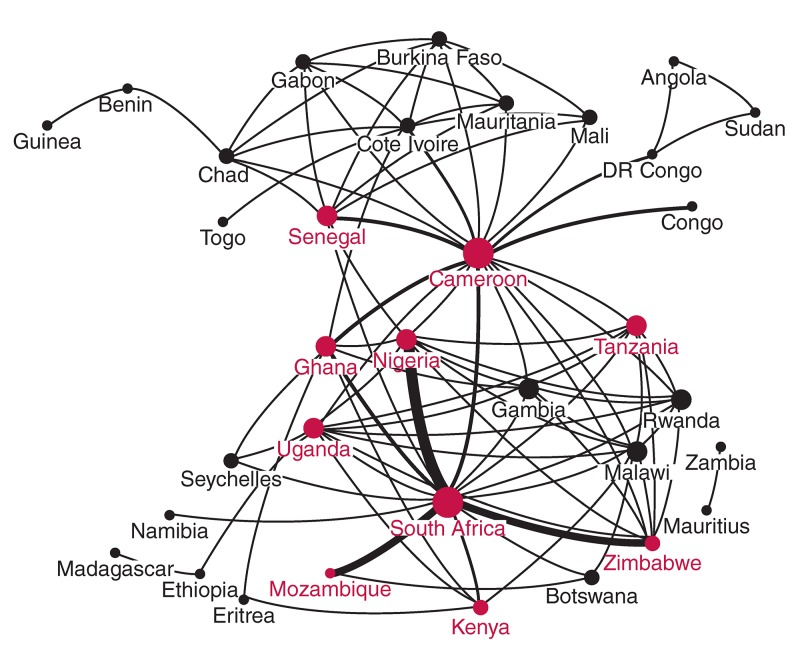
Network of cardiovascular research collaboration among countries in SSA. The 10 countries in red are those with the highest number of publications in cardiovascular research from 2005 to 2014. The width of the edges between countries reflects the extent of collaboration between the pair based on co-authorship.

The network of collaboration in CVR between countries in SSA can be visualised in Fig. 2. The network is based on the subset of publications in which more than one SSA country was present from authors’ affiliations. A total of 34 SSA countries were involved in at least one instance of collaboration out of the 47 countries in the region. The size of any vertex is proportional to the degree centrality, i.e. the number of countries with which that country has an instance of collaboration.

Cameroon had the highest degree centrality^18^ and eigenvector centrality (0.087), indicating a high degree of collaboration with other countries in the region, and playing a role as a boundary spanner linking the French-speaking countries with most of the English-speaking countries. The width of the edges connecting pairs of countries is proportional to the frequency of co-authorship or collaboration between these countries. The thickest edge occurs between South Africa and Nigeria, indicating the highest level of collaboration in CVR in the region. South Africa also has strong collaboration in CVR with Zimbabwe, Ghana and Mozambique.

## International collaboration and citation impact

Table 4 presents the 10-year trend in bibliometric outcomes for publications in CVR with authorship involving SSA. The table also compares research output and citation impact for publications with single-country authorship (SCP) and those with multi-country authorship (MCP). Overall, there was a consistent increase in the total number of publications and total number of citations each year from 2005 to 2014. The total number of publications over the 10-year period was similar for SCP and MCP, as shown by the publication ratio, with a slight increase from 0.7 in 2005 to 1.2 in 2014. Total citations rose from 20 in 2005 to 2 973 in 2014.

**Table 4:** Comparison of annual count and citations for single-country (SSA ) authored and multi-country (international) authored cardiovascular research publications

	*All publications*		*Single-country publications (SCP)*			Multi-country publications* (MCP)			*Comparisons (MCP/SCP)*		
*Year*	*Total publications*	*Total citations*	*Publications*	*Citations*	*Citation rate (CR)*	*Publications*	*Citations*	*Citation rate (CR)*	*Publication ratio*	*Citation ratio*	*CR ratio*
2005	88	20	51	4	0.1	37	16	0.4	0.7	4.0	5.5
2006	101	247	55	52	0.9	46	195	4.2	0.8	3.8	4.5
2007	108	547	58	122	2.1	50	425	8.5	0.9	3.5	4.0
2008	149	702	66	150	2.3	83	552	6.7	1.3	3.7	2.9
2009	156	1008	88	231	2.6	68	777	11.4	0.8	3.4	4.4
2010	163	1247	84	325	3.9	79	922	11.7	0.9	2.8	3.0
2011	164	1762	82	425	5.2	82	1337	16.3	1.0	3.1	3.1
2012	197	2104	96	498	5.2	101	1606	15.9	1.1	3.2	3.1
2013	218	2623	105	581	5.5	113	2042	18.1	1.1	3.5	3.3
2014	225	2973	101	729	7.2	124	2244	18.1	1.2	3.1	2.5
Total	1569	13233	786	3117	4.0	783	10116	12.9	1.0	3.2	3.3
Average	157	1,323	79	312	3.5	78	1012	11.1	1.0	3.4	3.6

*This refers to all multi-country publications that included at least one SSA country among the author affiliations.

There were substantial differences between the SCP and MCP in the citation frequency and citation rate (CR, number of citations per publication) throughout the period of analysis. On average, the annual citation frequency for MCP was 3.4 times higher than the annual citation frequency for SCP. Over 10 years, the total number of citations for the MCP was 10 116, compared to 3 117 for the SCP. Comparisons of citation rates for the two categories using the annual CR ratio showed that the CR for MCP was on average 3.6 times higher than that for SCP.

## Discussion

This study provides a 10-year picture of the range, volume and scientific impact of international collaboration in cardiovascular research in sub-Saharan Africa. The absence of published literature on this issue suggests that this is the first study that evaluates the comparative output of SSA countries in cardiovascular research and examines the patterns of co-authorship involving country-level affiliations within and outside SSA.

A study in 2005 on estimates of the global production in CVR showed that the scientific productivity of Africa (which included SSA and countries in North Africa) over the period 1995 to 2002 was the lowest of all the world regions, accounting for 0.3% of the global output in the field.^18^ Over the eight-year period, Africa produced 212 articles in the 38 journals included in the study, with a fairly constant average annual output of 27 articles per year.

In the present study, the annual output for SSA increased constantly from 88 in 2005 to 225 in 2014. The higher annual output in this study may be due to the difference in search methodology, in which published articles were not restrictively drawn from the 38 journals in the ‘Cardiac and Cardiovascular Systems’ category of ‘Journal Citation Report’ as was done in the study mentioned above.^18^ In general, there appears to be a gradual increase in research output in this field in SSA, although the contribution of the region to the global output remains very low.

Some of the reasons for the lower research productivity in SSA were mentioned above – lack of resources for research, the fewer number of research institutions, weak government support, and the dearth of skilled researchers in many fields of study.^5^,^6^ In addition, the pressure to publish in these countries is enormous, as promotion and tenure, institutional performance and external funding are strongly tied to publication counts.^19^,^20^

Collaboration in CVR between SSA and non-SSA countries shows similarities with the patterns reported for international collaborations across all scientific disciplines in Africa.^5^ South Africa remains the leading country both in terms of research output and the extent of collaboration with countries outside SSA. The top non-SSA countries involved in co-authorships in CVR with SSA countries are: USA, England, Italy, France, Germany and Canada. Most of these countries are reported in the top 10 of the world’s leading countries in terms of scientific output in cardiovascular research.^15^

The reasons underlying this pattern of collaboration in CVR are numerous. The strength of the national economies measured as gross domestic product has been shown to be positively associated with national research output.^18^ Although data on GDP was not utilised in the study, this association may partly explain the position of South Africa and Nigeria as the leading countries in CVR in the region.

Historical and political ties between countries may influence the extent of collaboration in research,^21^ as well as the dominant language of use in research. France is well known to be a critical partner with regard to international research collaboration in French-speaking Africa.^12^ The high degree centrality of Cameroon in the CVR network of SSA countries can be attributed to the presence of anglophone and francophone populations within the country. This unique characteristic of Cameroon makes it strategically important in creating and expanding collaborative research networks within SSA, essentially co-developing joint research projects with groups of cardiovascular researchers in both English- and French-speaking countries in the region. Such networks are critically important for improved rigour and cost-effectiveness of research endeavours in SSA, with the advantage of multi-country study designs and the possibility of concurrent policy engagement strategies in different countries using common evidence to influence health policy and practice aimed at reducing cardiovascular morbidity and mortality rates in the region.

International collaborations in SSA are strongly linked to the source of research funding from countries outside the region. A large number of research articles in public health with African authorship can be linked to the grants awarded for projects in specific countries from the major funding agencies, such as USAID, the UK Medical Research Council, and the Wellcome Trust.5 In addition, research training opportunities for students from SSA in developed countries provide avenues for collaboration with researchers in the host universities.^22^

Nigeria presents a unique case of low international collaboration in spite of a relatively high CVR output compared to most countries in the region. This may be due to the availability of greater local funding for CVR and the relatively higher number of universities with medical faculties compared to most other SSA countries. The motivation for researchers in Nigeria to publish for career advancement purposes may positively influence the national research output in this field without a significant influence from international funding and collaboration2^23^

The extent of collaboration within SSA is very limited compared to the level of collaboration with other non-SSA countries. This pattern has been observed with data for all of the scientific output of the region.^5^,^8^,^9^,^24^ Some studies have found lower levels of collaboration between countries in West Africa compared with their individual levels of collaboration with France.^25^,^26^ South Africa and Nigeria, which account for the bulk of the cardiovascular research output in SSA, partly due to the size of their cardiovascular research communities, are best placed to serve as strategic hubs for promoting and coordinating collaboration in this field.

In South Africa, the Hatter Institute of Cardiovascular Research in Africa, located at the University of Cape Town, is working to facilitate national and international research collaborations to combat cardiovascular disease in Africa.^27^ Two key multinational collaborative projects underway at the institute include: (1) the Pan-African Pulmonary Hypertension Cohort (PAPUCO) study, which aims to describe the epidemiology of pulmonary hypertension in patients from 10 African countries,^28^ and (2) the THESUS-HF survey, which focuses on the causes, treatment and outcome of acute heart failure in patients across nine African countries.^29^ These large, collaborative studies reflect real intent and progress in building regional networks that fosters international collaboration in cardiovascular research.

In the last few years, there has been a revival of the Pan-African Society of Cardiology (PASCAR), leading to improved networking among researchers and clinicians involved in cardiovascular research across Africa.^30^ Some of the major multinational collaborative research projects that involve PASCAR include: the Awareness Surveillance Advocacy Prevention (ASAP) programme,^31^ the ASTRAL study targeted at controlling hypertension,^32^ the IMPI trial for the management of tuberculous pericarditis,^33^ and the Human Heredity and Health in Africa (H3Africa) initiative.^34^ These large initiatives are transforming the landscape of cardiovascular research in Africa, and the resulting enhanced capacity of African scientists will be evident from increased research productivity in this field and the contributions to knowledge that will ultimately benefit the continent.

A benefit of international collaboration in CVR is seen in the greater citation impact that resulted from publications with multi-country authorship compared with that from publications with single-country authorship. Despite similar research outputs over the 10-year period, the multi-country publications resulted in over three times the number of citations garnered by the single-country publications. While no analysis was done on the range of journals in which the articles were published and how these could influence citation rates, this finding likely reflects the greater possibilities for dissemination and utilisation of the knowledge generated through the networks associated with the multiple authors and affiliated institutions in the countries involved. These results are similar to those reported by other authors.^35^,^36^

## Limitations

This study has a number of limitations. First, the analysis involved publications contained in the Web of Science and therefore excludes the numerous journals not indexed, particularly journals in countries in SSA. Web of Science was used in this study because it provided the affiliations of all the authors listed in each record for the time period under consideration, without which the study would not have been possible. Second, the use of co-authorship as a form of collaboration is limited by the assumption of interaction between all pairs of authors. The impact of co-authorship on future collaboration is not well understood and requires further study. Lastly, the use of citation measures as evidence of scientific impact has its limitations,particularly with regard to the journals and databases used, self-citation issues, and the unequal access to articles in different journals due to the use or otherwise of an open-access policy. In spite of these limitations, this study utilised a reliable data source and methodologies that provided valuable evidence on connectedness of countries involved in cardiovascular research in SSA.

## Conclusion

This study provides evidence on the state and scientific impact of national-level collaboration in cardiovascular research in SSA. It reveals the very low, but growing, research output and collaboration in this field from the region, even as the burden of cardiovascular disease continues to rise in SSA. Research institutions and national governments in SSA need to pro-actively work to build effective cardiovascular research networks that include multiple countries in the region, as a means of developing capacity in this field and improving the quality and volume of research in cardiovascular disease prevention and care. Creating and strengthening international research networks in SSA is critical if the growing challenge of a rising cardiovascular disease burden in the region is to be addressed effectively.
